# Haemodynamic correlates of bilateral 6 Hz transcranial alternating current stimulation during working memory enhancement revealed by fNIRS

**DOI:** 10.3389/fnins.2026.1788696

**Published:** 2026-05-11

**Authors:** Zhenghao Dong, Yingli Bi, Han Yang, Feilong Zhu, Jincheng Li, Mingxue Fan, Shiyan Wang, Zunke Gong

**Affiliations:** 1 Xuzhou Rehabilitation Hospital Affiliated to Xuzhou Medical University, Xuzhou, China; 2 Xuzhou Medical University, Xuzhou, China; 3 Xuzhou Rehabilitation Hospital, Xuzhou, China; 4 Department of Rehabilitation Medicine, Xuzhou Center Hospital, Xuzhou, China; 5 Department of Rehabilitation Medicine, The First Affiliated Hospital with Nanjing Medical University, Nanjing, China; 6 School of Sports Medicine and Rehabilitation, Beijing Sport University, Beijing, China

**Keywords:** 6 Hz theta band, dorsolateral prefrontal cortex (DLPFC), functional near-infrared spectroscopy (fNIRS), transcranial alternating current stimulation (tACS), working memory (N-back)

## Abstract

**Introduction:**

Theta-band transcranial alternating current stimulation (tACS) has been proposed to enhance working memory (WM) by entraining endogenous oscillations, yet the haemodynamic signatures accompanying theta-tACS–related WM gains remain unclear. We investigated whether 6 Hz tACS modulates prefrontal task-evoked haemodynamic responses during WM and whether such changes relate to behavioral improvement.

**Methods:**

In a randomized, single-blind, sham-controlled design, healthy adults (18–30 years) were allocated to 6 Hz tACS or sham (initially *n* = 25 per group). Stimulation (1mA peak-to-peak, 20min, 30-sramp-up/down) was delivered bilaterally over the dorsolateral prefrontal cortex (DLPFC; F3/F4). Participants completed the Digit Span Test and N-back tasks (1-, 2-, 3-back) before and after stimulation. During N-back performance, fNIRS recorded bilateral DLPFC signals. Task-evoked oxygenated hemoglobin (HbO) β coefficients were estimated using a general linear model. Group differences in pre–post changes were tested using within-/between-group analyses and ANCOVA, with brain–behavior coupling assessed via Spearman correlation.

**Results:**

After fNIRS quality control, 44 participants were included in the final analysis; six participants were excluded because more than 30% of channels were identified as low quality. Relative to sham, 6 Hz tACS produced greater improvements in backward Digit Span and total Digit Span. In the N-back task, accuracy improved selectively under the high-load 3-back condition (between-group change: [F_(1.40)_ = 12.29, p_adj = 0.0034, η^2^*p* = 0.24)], whereas reaction time showed no significant between-group differences. fNIRS revealed post-stimulation increases in left DLPFC HbO-β during 3-back, with a significant between-group β-change at one channel [(F_(1.40)_ = 10.69, p_adj = 0.035)]. Within the 6 Hz group, 3-back accuracy gains correlated positively with β-change in the left DLPFC (ρ = 0.44, p_adj = 0.039).

**Conclusion:**

Bilateral 6 Hz tACS selectively enhances high-load WM accuracy and is accompanied by increased task-evoked haemodynamic activation in the left DLPFC. The observed brain–behavior coupling suggests that theta-frequency neuromodulation may facilitate executive control under high cognitive demand via strengthened prefrontal neurovascular responses.

## 1 Introduction

Working memory (WM) is commonly defined as a capacity-limited cognitive system that temporarily maintains and manipulates task-relevant information. Such information is often composed of activated components from long-term memory and is selected and updated under attentional control, thereby supporting complex cognitive activities such as comprehension, learning, and reasoning (Baddeley, 2000; Brooks et al., 2022; Burgess et al., 2011). Evidence indicates that declines in WM are closely associated with weakened attentional control and reduced fluid intelligence, contributing to age-related deterioration in multiple cognitive functions and impairments in everyday functioning (Liao et al., 2024; McAlister and Schmitter-Edgecombe, 2016). Therefore, it is imperative to identify effective methods and strategies to enhance working memory.

From a neurophysiological perspective, converging evidence from animal electrophysiology and human neuroimaging indicates that neurons in the dorsolateral prefrontal cortex (DLPFC) exhibit selective, persistent firing during the delay period, and that recurrent excitation and lateral inhibition microcircuits—modulated by dopaminergic signaling—provide a critical cellular basis for the active maintenance of working memory (Goldman-Rakic, 1995). Meanwhile, working memory is not confined to a single region but relies on distributed representations within networks such as the prefrontal–parietal system, which support the selective encoding of task-relevant information (D'Esposito and Postle, 2015). Building on this, the “activity-silent” model proposes that some memory functions can be maintained via transient changes in synaptic weights, remaining in low-firing or even non-firing states, and that information encoding, reactivation, and readout are supported by oscillatory bursts in the gamma and beta bands (Stokes, 2015). Collectively, these findings suggest that working memory capacity and plasticity are shaped not only by local cortical excitability but also by network connectivity strength and cross-frequency oscillatory coordination (Constantinidis and Klingberg, 2016).

Against this mechanistic backdrop, an increasing number of studies have attempted to directly modulate relevant neural circuits using non-invasive brain stimulation (NIBS) to enhance working memory. Major NIBS approaches include transcranial magnetic stimulation (TMS), transcranial direct current stimulation (tDCS), and transcranial alternating current stimulation (tACS) (Polanía et al., 2018). Among these techniques, tACS has shown particular promise due to its ability to modulate oscillatory neural dynamics in a frequency- and phase-specific manner. tACS delivers low-frequency sinusoidal alternating currents that can entrain endogenous brain oscillations via phase synchronization (phase entrainment), enabling more precise modulation of neural rhythms across both temporal and frequency domains (Klink et al., 2020). Prior work suggests that tACS exerts frequency-specific effects on working memory: for instance, alpha-band stimulation can benefit short-term memory processing (Meiron and Lavidor, 2014), whereas gamma-band stimulation may accelerate response speed in operational tasks (Santarnecchi et al., 2016). However, theta-band stimulation should not be regarded as underexplored in the working-memory field. Rather, theta-tACS has become one of the most extensively discussed stimulation frequencies because theta oscillations are closely linked to executive control, memory updating, and frontoparietal coordination. Recent reviews have summarized a substantial body of tACS studies on working memory, many of which specifically involved theta-frequency protocols, and quantitative syntheses further suggest that tACS effects on working memory are shaped by stimulation parameters, montage, and task characteristics (Senkowski et al., 2022; Al Qasem et al., 2022; Nissim et al., 2019). Representative studies have also shown that theta-frequency stimulation can modulate neural oscillations and behavioral performance. For example, 6 Hz stimulation may enhance inter-regional neural coupling and network coordination through phase alignment, thereby modulating prefrontal–parietal theta networks and improving cognitive efficiency (Riddle et al., 2020; Van Hoornweder et al., 2025). Recent reviews further suggest that theta-range tACS can influence oscillatory dynamics and cognitive performance, particularly under high task demands (Barzegar et al., 2025). In addition, Hosseinian et al. demonstrated that phase-synchronized 6 Hz tACS in healthy adults significantly enhanced prefrontal theta power and inter-regional synchrony, accompanied by improved working-memory performance (Hosseinian et al., 2021). Against this background, the key gap is not whether theta-tACS is relevant to working memory, but that its cortical haemodynamic signatures—especially task-evoked prefrontal responses measured with fNIRS—remain insufficiently characterized.

Nevertheless, much of the existing tACS literature has relied primarily on EEG to evaluate stimulation effects. Such electrophysiological measures have limited spatial resolution and are susceptible to artifacts, making it difficult to precisely characterize the impact of tACS on prefrontal cortical activation. fNIRS is an emerging neuroimaging modality that tracks haemodynamic responses by exploiting differential absorption of near-infrared light by oxygenated hemoglobin (HbO) and deoxygenated hemoglobin (HbR), thereby providing an indirect index of local neural activity (Scholkmann et al., 2014). Prior research indicated that, via neurovascular coupling, fNIRS can capture oxygenation changes triggered by neural activity and is well suited for task-based paradigms (Ferrari and Quaresima, 2012). Compared with EEG, fNIRS offers greater spatial specificity for monitoring activation in prefrontal regions involved in working memory, which may facilitate the identification of tACS-related haemodynamic alterations within the prefrontal cortex (McKendrick et al., 2015; Tak et al., 2016).

In summary, the present study applied 6 Hz tACS over the bilateral DLPFC in healthy adults and combined fNIRS with behavioral assessment to systematically examine its modulatory effects on prefrontal haemodynamic responses and working memory performance. Participants completed two working memory tasks before and after the intervention: the Digit Span Test (forward, backward, and total) and the N-back task (1-back, 2-back, and 3-back). During N-back performance, bilateral DLPFC fNIRS signals were recorded, and task-related cortical activation was indexed using β values. The primary aims were: (1) to determine whether 6 Hz tACS improves working memory performance in healthy individuals; and (2) to explore the mechanisms by which 6 Hz tACS modulates prefrontal neural activity and haemodynamic responses by comparing pre- and post-intervention changes in fNIRS β values.

## 2 Materials and methods

### 2.1 Study design and setting

**Figure 1:**
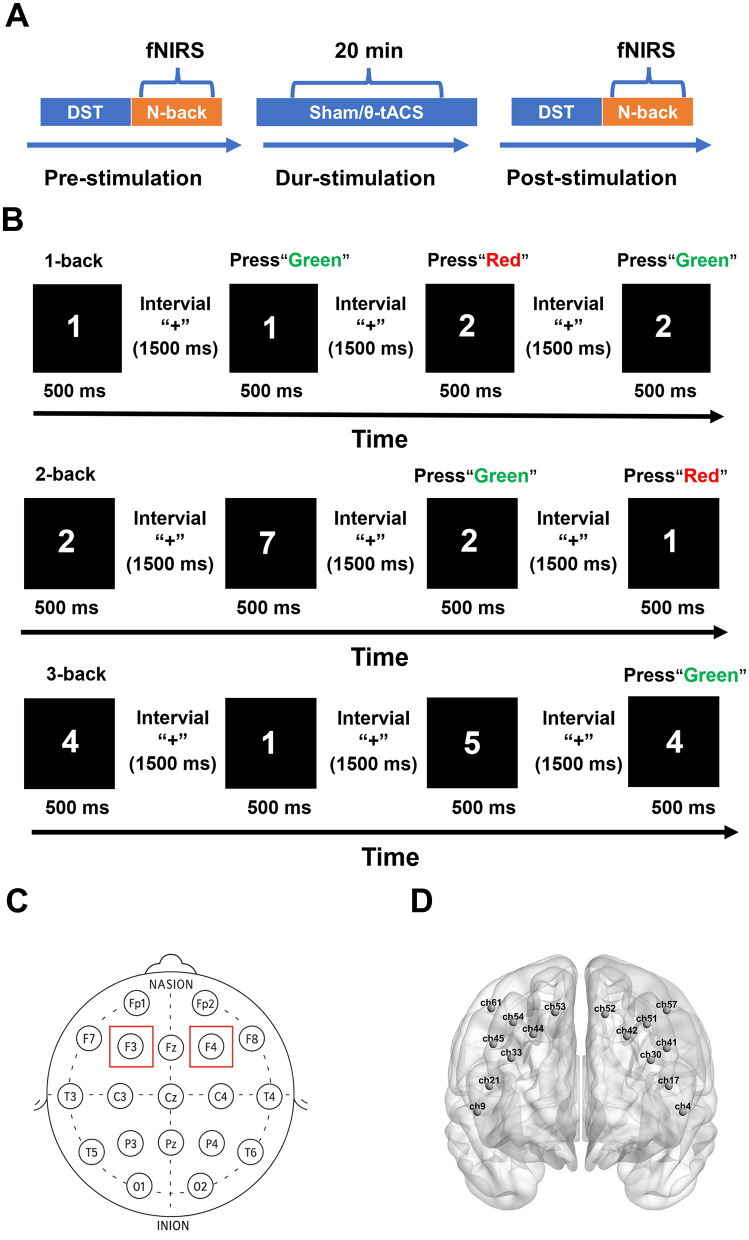
**(A)** Study flow diagram illustrating the experimental protocol. **(B)** Schematic illustration of the 1-back, 2-back, and 3-back tasks. **(C)** Locations of tACS electrodes positioned at F3 and F4 according to the international 10–20 system. **(D)** fNIRS channel localization and regions of interest over the bilateral dorsolateral prefrontal cortex.

This study adopted a randomized, single-blind, sham-controlled design. All procedures were conducted in an electromagnetically shielded room. As shown in Figure 1A, the protocol comprised three phases. In the pre-stimulation phase, participants completed the Digit Span Test and the N-back task (1-back, 2-back, and 3-back) in sequence. In the during-stimulation phase, participants received 20 min of tACS without performing any behavioral tasks. In the post-stimulation phase, the same assessments as in the pre-stimulation phase were repeated.

### 2.2 Participants

Healthy volunteers aged 18–30 years were recruited for this study. All participants were right-handed and had normal or corrected-to-normal vision. Individuals were excluded if they reported any history of neurological or psychiatric disorders, major physical illness, or substance (drug or alcohol) dependence. Participants were also excluded if they had contraindications to transcranial electrical stimulation (e.g., intracranial metal implants, pregnancy).

Sample size was estimated using G^*^Power based on parameters reported by (Ke et al. 2019). A two-tailed test was adopted with a significance level of α = 0.05 and statistical power of 0.80, indicating that at least 20 participants were required per group. To account for an anticipated dropout rate of 20%, the target sample size was set at 25 participants per group (total *N* = 50).

Fifty participants were randomly allocated to either the 6 Hz tACS group or the sham group (n = 25 per group). Single blinding was supported by the sham stimulation procedure, in which only the initial ramp period was applied to induce a typical tingling sensation (Park et al., 2025). Written informed consent was obtained from all participants prior to participation.

### 2.3 Intervention

#### 2.3.1 Control group (sham)

In the sham condition, stimulation was delivered only during the initial 30-s ramp-up to mimic typical cutaneous sensations and support single-blinding (Park et al., 2025). The stimulation phase lasted 20 min, during which no behavioral tasks were performed.

#### 2.3.2 Experimental group (6 Hz tACS)

tACS was delivered using a transcranial alternating current stimulator (model A6201-2; Jiangxi Weizhiguang Medical Technology Co., Ltd., China; product registration no. 2025209009). Theta-frequency stimulation (6 Hz) was applied. For the active condition, stimulation intensity was set at 1 mA peak-to-peak and delivered for 20 min, with a 30-s ramp-up and a 30-s ramp-down at the beginning and end to reduce stimulation-related discomfort. Square electrodes (5 cm × 5 cm) were positioned at F3 and F4 according to the international 10–20 system (Figure 1C), targeting the bilateral dorsolateral prefrontal cortex. Stimulation-related sensations were assessed by verbal inquiry during and immediately after stimulation and recorded by the experimenter.

### 2.4 Behavioral assessment procedures

**Digit Span Test**. The Digit Span Test (DST) is a classic tool for assessing working memory capacity and attentional control (Egeland et al., 2025). Forward digit span primarily indexes short-term maintenance, whereas backward digit span is more sensitive to active manipulation and executive control; these components provide complementary information (Heled and Levi, 2025; Reed and Hillmann, 2025). Digits were presented by an examiner at approximately 1 s per digit. For the forward condition, sequences began with three digits and participants repeated them in the same order; for the backward condition, sequences began with two digits and participants recalled them in reverse order. Testing terminated when two consecutive errors occurred at the same sequence length; the score for each condition was the longest sequence length recalled correctly, and the DST total score was calculated as the sum of forward and backward scores (Nisha et al., 2025; Ramesh et al., 2025). (Can et al., 2025; Cheng et al., 2024).

**N-back task**. The N-back task was originally proposed by Kirchner and is one of the most widely used paradigms for assessing working memory (Kirchner, 1958; Owen et al., 2005). Its performance indices are considered valid and reliable markers of working memory ability (Jaeggi et al., 2010). In this study, three load conditions were administered (1-back, 2-back, and 3-back; Figure 1B). Participants were required to judge whether the current digit matched the digit presented one, two, or three trials earlier, respectively. They pressed the green button for a match and the red button for a non-match, and were instructed to respond as quickly and accurately as possible. Each block comprised 15 digits, and each load condition consisted of three blocks. In the 1-back condition, responses began from the second digit, yielding 14 evaluable judgements per block; in the 2-back condition, responses began from the third digit, yielding 13 evaluable judgements; and in the 3-back condition, responses began from the fourth digit, yielding 12 evaluable judgements. Within the valid evaluable trials of each block, the target-to-non-target ratio was 4:10 in the 1-back condition, 4:9 in the 2-back condition, and 4:8 in the 3-back condition. Baseline periods were included before and after each block, during which a fixation cross (“+”) was displayed. Each digit was presented for 500 ms, followed by a 1,500 ms interstimulus interval. All participants began with 1-back practice. When practice accuracy reached ≥80%, they were considered to have mastered the task rules and then proceeded sequentially to 2-back and 3-back practice and formal testing. During the experiment, participants were instructed to remain seated and minimize head and body movements. The testing environment was kept quiet and well lit to ensure data quality (Ayaz et al., 2012). Accuracy and reaction time (RT) were recorded as behavioral indices, as both have been widely used to capture workload-related changes in working memory performance and prefrontal functional states (Antonenko et al., 2016; Gateau et al., 2018; Heinzel et al., 2017; McKendrick et al., 2016).

### 2.5 Outcomes and data collection

#### 2.5.1 Primary outcome

The primary outcome was N-back task accuracy, defined as the proportion of correct responses relative to the total number of trials for each load condition (1-back, 2-back, and 3-back) (Jacola et al., 2014).

#### 2.5.2 Secondary outcomes

Secondary behavioral outcomes included N-back reaction time (RT) and Digit Span Test (DST) performance. RT was calculated as the mean response time across correct trials within each load condition; trials with RT < 200 ms or >1,500 ms were excluded (Steinberg et al., 2022). DST outcomes comprised forward digit span, backward digit span, and the total score (forward + backward) (Egeland et al., 2025; Heled and Levi, 2025; Reed and Hillmann, 2025; Nisha et al., 2025; Ramesh et al., 2025). (Can et al., 2025; Cheng et al., 2024).

### 2.6 fNIRS data acquisition and processing

fNIRS data were acquired using a BS-2000 near-infrared spectroscopy system (Wuhan Zilian Hongkang Technology Co., Ltd., China) with dual-wavelength laser diodes (690 nm and 830 nm) and a sampling rate of 20 Hz. The montage comprised 22 sources and 20 detectors, yielding 67 channels with a 3-cm source–detector distance. Optodes were arranged according to the international 10–20 system, with source S2 corresponding to Fpz. A 3D digitiser (NirMap) was used to record standard reference points (Nz, Cz, AL, RL) and the spatial coordinates of all optodes. Spatial registration was performed using NIRS-SPM to project channels onto the cortical surface and map them to Brodmann areas. Channels were grouped into 14 regions of interest (ROIs), with the bilateral dorsolateral prefrontal cortex (DLPFC) defined as the primary ROI (Figure 1D).

Task-evoked β estimation and GLM construction were performed using the Homer2 toolbox combined with in-house MATLAB scripts. The processing pipeline was as follows: (1) raw data were converted from csv to nirs format using csv2nirs; (2) channel-level quality control was conducted by calculating the coefficient of variation (CV), and channels with CV > 25% were regarded as low quality and excluded. At the participant level, datasets were excluded from the final analysis when more than 30% of channels were identified as low quality, leaving insufficient usable channels for reliable estimation of task-evoked HbO β values in the bilateral DLPFC; (3) light intensity signals were converted to optical density (OD); (4) motion artifacts were identified and corrected using spline interpolation to minimize head-motion contamination; (5) a 0.01–0.1 Hz band-pass filter was applied to remove low-frequency drifts (e.g., slow physiological fluctuations) and high-frequency physiological noise (e.g., cardiac and respiratory components); and (6) OD signals were converted to concentration changes of HbO and HbR using the modified Beer–Lambert law, followed by moving-average smoothing and baseline correction.

A general linear model (GLM) was then constructed for statistical analysis. The design matrix included the task regressor(s) and a constant term. Task regressors were generated by convolving the task time series (boxcar function) with a canonical haemodynamic response function (HRF). As low-frequency drifts and motion artifacts had been addressed during preprocessing (filtering and spline interpolation), no additional drift terms or motion parameters were included as covariates in the regression model. Finally, channel-wise β coefficients were estimated using least-squares fitting to quantify task-related cortical activation strength.

### 2.7 Statistical analysis

This study performed statistical analyses of baseline demographic characteristics, behavioral outcomes, and fNIRS β values in the 6 Hz intervention group and the sham group. For continuous variables, normality was assessed using the Shapiro–Wilk test, and homogeneity of variance was examined using Levene's test. Normally distributed continuous variables are presented as mean ± standard deviation (SD), whereas non-normally distributed continuous variables or ordinal data are presented as median (interquartile range, IQR). Categorical variables are presented as frequency (percentage, %).

For baseline demographic characteristics, independent-samples *t*-tests were used for normally distributed continuous variables, Mann–Whitney U tests were used for non-normally distributed continuous variables, and χ^2^ tests were used for categorical variables. Welch's *t*-test was applied to compare between-group differences in baseline behavioral and neuroimaging measures. Paired-samples *t*-tests were then used to examine within-group pre–post changes in behavioral and neuroimaging measures.

To further evaluate intervention effects, change scores (post–pre) in behavioral and neuroimaging measures were entered as dependent variables in a one-way analysis of covariance (ANCOVA), with group as the fixed factor and age and sex as covariates; pairwise comparisons were subsequently conducted based on estimated marginal means (EMMs). In addition, to examine the relationship between neural activation changes and behavioral improvement, Spearman's rank correlation was used to test associations between changes in N-back accuracy and changes in β values in the significant channels.

All analyses were performed in R (version 4.3.1) and MATLAB 2014a (MathWorks, Natick, MA). Statistical significance was set at 0.05 (two-tailed). To control the family of Type I errors due to multiple comparisons, all *p* values were adjusted using the Benjamini–Hochberg (BH) procedure under the false discovery rate (FDR) framework.

## 3 Results

### 3.1 Baseline characteristics

Of the 50 participants initially enrolled, 44 were included in the final analysis after fNIRS quality control. Six participants were excluded because more than 30% of channels were identified as low quality. The final sample comprised 22 participants in the 6 Hz tACS group and 22 participants in the sham group. Baseline demographic variables were compared between the 6 Hz group and the sham group. An independent-samples *t*-test indicated that the mean age was 21.73 ± 2.45 years in the 6 Hz group and 22.86 ± 1.91 years in the sham group, with no significant between-group difference (*t* = −1.72, *p* = 0.094). Sex distribution, assessed using the χ^2^ test, also did not differ significantly between groups (6 Hz: 16 females [72.7%], 6 males [27.3%]; sham: 12 females [54.5%], 10 males [45.5%]; χ^2^ = 0.89, *p* = 0.347). Overall, the two groups were comparable in baseline demographic characteristics.

### 3.2 Digit span test

**Figure 2:**
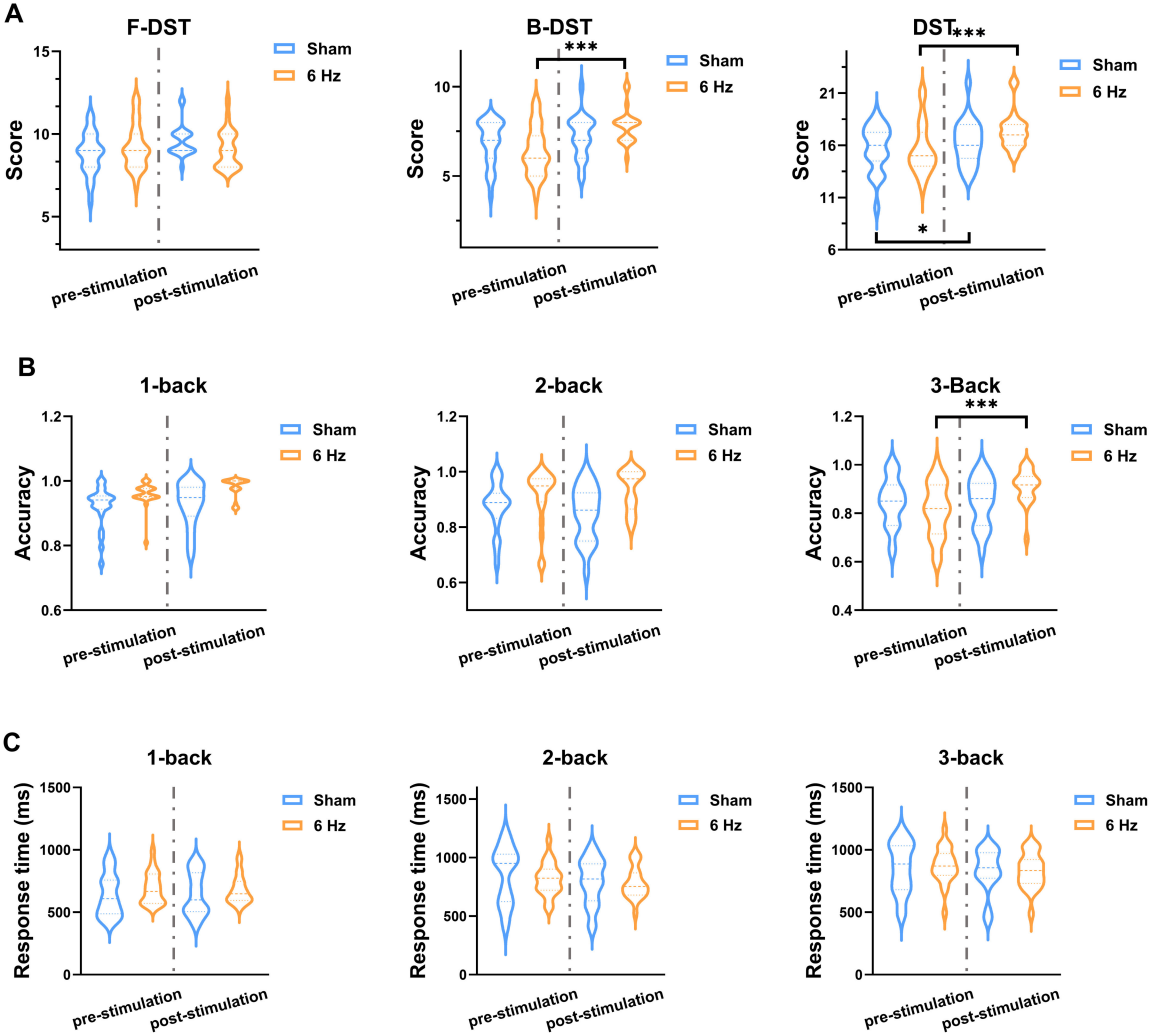
Behavioral performance before and after stimulation. **(A)** Digit Span Test results. **(B)** N-back task accuracy. **(C)** N-back reaction time. Error bars represent standard deviations. * and *** Indicate statistical significance levels based on FDR-corrected *p*-values (p_adj): * p_adj < 0.05, ** p_adj < 0.01, *** p_adj < 0.001.

The behavioral results, including Digit Span Test performance, N-back accuracy, and reaction time, are presented in Figure 2. Within-group paired t-tests showed that, compared with pre-stimulation, the 6 Hz group demonstrated significant post-stimulation increases in backward digit span and total score (backward: *t* = 6.89, df = 21, p_adj < 0.001; total: *t* = 5.63, df = 21, p_adj < 0.001). In the sham group, only the total score increased significantly after stimulation (*t* = 2.52, df = 21, p_adj = 0.039). Between-group comparisons indicated no significant differences at baseline in forward, backward, or total Digit Span scores. When comparing the magnitude of change (post–pre) between groups, significant between-group differences were observed for backward digit span [(F_(1.40)_ = 14.29, p_adj = 0.0015, ${{\text{η}}^{2}}_{\text{P}}$ = 0.26)] and total score [(F_(1.40)_ = 6.67, p_adj = 0.0204, ${{\text{η}}^{2}}_{\text{P}}$ = 0.14)]. *Post-hoc* analyses based on estimated marginal means showed that the 6 Hz group exhibited greater improvements than the sham group in backward digit span (*t* = 3.78, p_adj = 0.0015) and total score (*t* = 2.58, p_adj = 0.0204).

### 3.3 N-back accuracy

Within-group paired *t*-tests showed that, relative to pre-stimulation, the 6 Hz group exhibited a significant increase in accuracy in the 3-back condition (*t* = 4.82, df = 21, p_adj < 0.001), whereas no significant changes were found in the 1-back or 2-back conditions. In the sham group, accuracy did not change significantly in any load condition. Baseline accuracy did not differ significantly between groups in the 1-back, 2-back, or 3-back conditions. When comparing the magnitude of change between groups, a significant between-group difference was observed only for the 3-back condition [(F_(1.40)_ = 12.29, p_adj = 0.0034, ${{\text{η}}^{2}}_{\text{P}}$ = 0.24)]. *Post-hoc* analysis indicated that the improvement in 3-back accuracy was significantly greater in the 6 Hz group than in the sham group (*t* = 3.51, p_adj = 0.0034).

### 3.4 N-back reaction time

Within-group paired *t*-tests indicated that reaction time did not change significantly from pre- to post-stimulation in either group across the three task loads. Baseline reaction time also did not differ significantly between groups. Between-group comparisons of change scores showed no significant group differences in reaction time for 1-back [(*F*_(1.40)_ = 0.35, p_adj = 0.559)], 2-back [(*F*_(1.40)_ = 0.72, p_adj = 0.559)], or 3-back [(*F*(1. 40) = 0.41, p_adj = 0.559)].

### 3.5 fNIRS β values

**Figure 3:**
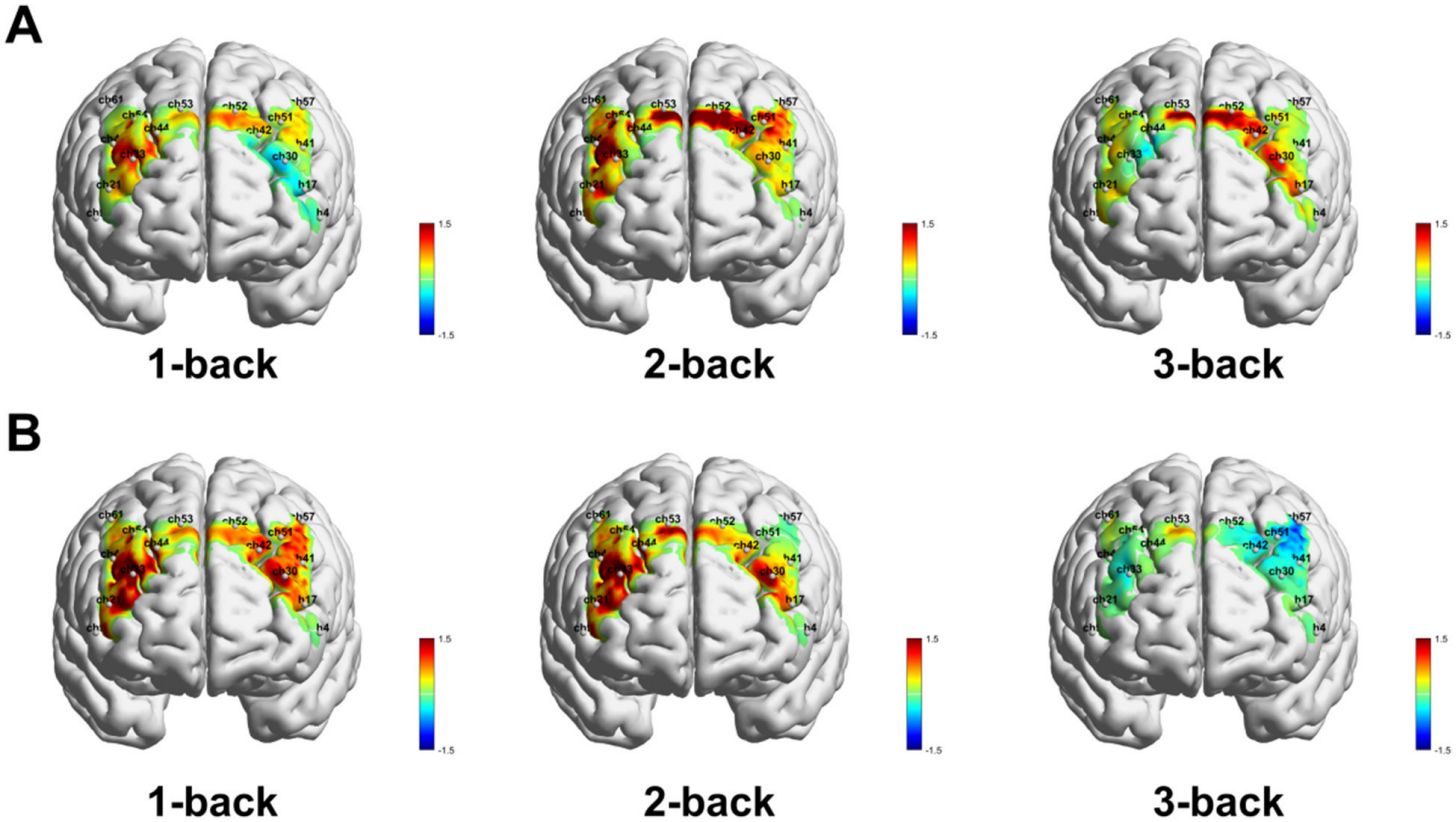
Schematic representation of paired *t*-test results for fNIRS activation. **(A)** 6 Hz tACS group.**(B)** Sham group.

**Figure 4:**
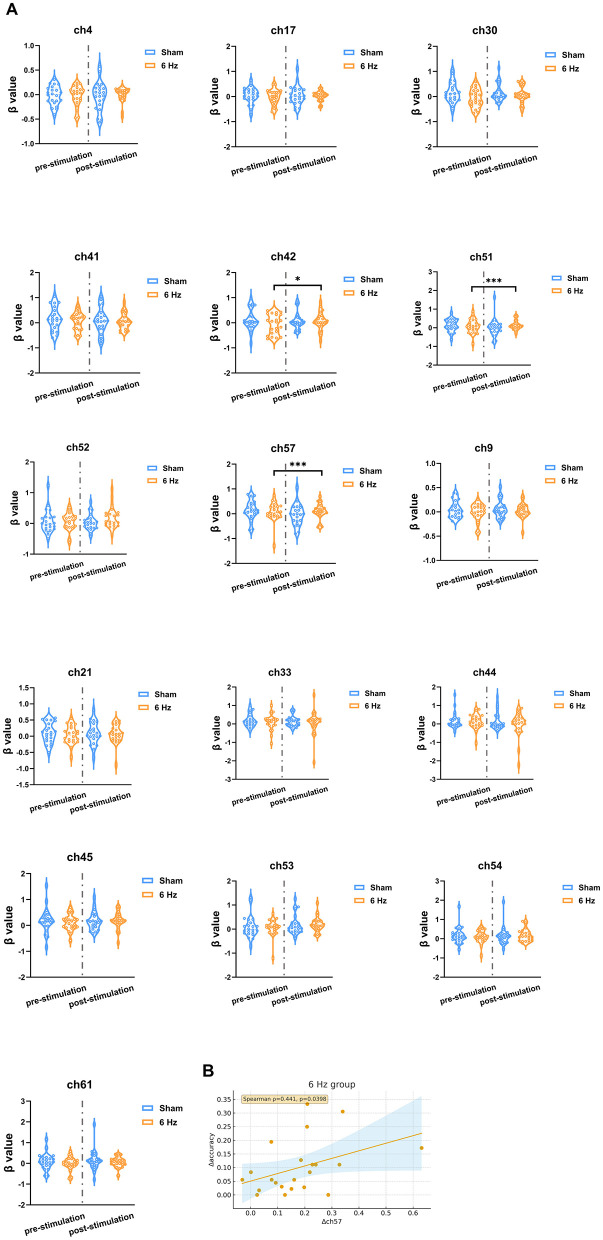
**(A)** Channel-wise comparisons of task-evoked HbO β values before and after stimulation in the 3-back condition. **(B)** Scatter plot showing the correlation between changes in 3-back accuracy and changes in β values in the left DLPFC. * and *** Indicate statistical significance levels based on FDR-corrected *p*-values (p_adj): * p_adj < 0.05, ** p_adj < 0.01, *** p_adj < 0.001.

Within-group paired *t*-tests showed that, during the 3-back task, the 6 Hz group demonstrated significant post-stimulation increases in activation within the left dorsolateral prefrontal cortex (DLPFC-L) (Figure 3), primarily in channels 42 (*t* = 3.21, df = 21, p_adj = 0.022), 51 (*t* = 5.52, df = 21, p_adj < 0.001), and 57 (*t* = 5.80, df = 21, p_adj < 0.001) (Figure 4A). In contrast, the sham group showed no significant pre–post changes in β values across channels in any of the three tasks. Baseline channel-wise β values did not differ significantly between groups in the 1-back, 2-back, or 3-back conditions. When comparing the magnitude of β-value change between groups, a significant between-group difference was observed for channel 57 in the 3-back condition [(*F*_(1.40)_ = 10.69, p_adj = 0.035). *Post-hoc* analysis indicated that the β-value increase at channel 57 was significantly greater in the 6 Hz group than in the sham group (*t* = 3.27, p_adj = 0.002).

### 3.6 Correlation analysis

Within the 6 Hz group, Spearman rank correlation analysis was performed to examine the association between the improvement in 3-back accuracy and the change in β value at channel 57. A significant positive correlation was found (ρ = 0.44, p_adj = 0.039) (Figure 4B).

### 3.7 Tolerability

No serious adverse events were observed in either group. Based on verbal inquiry, transient phosphene-like sensations were reported by 4 of 22 participants in the active group and 2 of 22 participants in the sham group, whereas mild tingling was reported by 10 of 22 participants in the active group and 5 of 22 participants in the sham group. All reported sensations were transient and resolved after stimulation.

## 4 Discussion

### 4.1 Principal findings

This study examined the modulatory effects of 6 Hz tACS targeting the bilateral DLPFC on working memory, integrating behavioral measures with haemodynamic assessment. Behaviourally, participants receiving 6 Hz tACS showed greater improvements in backward Digit Span and total Digit Span relative to sham, suggesting an influence on maintenance and reorganization processes. In the N-back task, accuracy increased selectively under the highest-load 3-back condition, indicating that the stimulation benefit emerged when continuous updating and interference monitoring demands were greatest. Notably, changes in 3-back performance were positively associated with task-evoked haemodynamic responses in the left DLPFC, consistent with load-dependent recruitment of prefrontal control resources in the post-stimulation session. Taken together, these findings support the interpretation that bilateral 6 Hz tACS may facilitate high-load WM performance, potentially via augmenting task-related neurovascular responses in prefrontal cortex.

### 4.2 Behavioral effects and proposed mechanisms

Reinhart and Nguyen showed that individualized theta-frequency HD-tACS improved change-detection accuracy with minimal effects on reaction time in healthy younger adults and, critically, restored performance in healthy older adults, consistent with enhanced processing efficiency (Reinhart and Nguyen, 2019). In line with this, our data indicate that 6 Hz tACS facilitates working-memory performance in a load-selective manner, with benefits emerging primarily under high-load conditions that demand intensive updating and interference monitoring. By contrast, Debnath et al. reported that 5 Hz tACS under high load shortened reaction time without significantly altering accuracy in healthy adults (Debnath et al., 2024). Together, these findings suggest that stimulation frequency may differentially influence working-memory subprocesses. In the present study, 6 Hz tACS predominantly increased accuracy rather than reducing reaction time, implying stronger effects on representational fidelity and executive control than on motor execution. Mechanistic support for a network-level account comes from theta HD-tACS work showing improved task performance accompanied by increased fronto–parietal synchrony on resting-state EEG (Zhang et al., 2022). Given that accuracy is more sensitive to maintenance stability, updating quality, and interference inhibition, whereas reaction time is additionally constrained by speed–accuracy trade-offs and non-decision components (Hosseinian et al., 2021), it is plausible that under 3-back—when DLPFC-driven monitoring and updating demands peak−6 Hz tACS enhances prefrontal theta-supported top-down control and stabilizes task-relevant representations, manifesting chiefly as accuracy gains (Mirjalili et al., 2022). Overall, the behavioral effects of 6 Hz tACS appear stage- and load-selective, with the most evident benefit when sequential maintenance and dynamic updating demands are high (Diedrich et al., 2025; Hou et al., 2025). Prior evidence consistently indicates that the DLPFC does not merely subserve “storage”, but plays a central role in executive control during working memory, including stabilizing goal representations, suppressing distractors, and exerting top-down regulation during updating and manipulation (Jensen and Tesche, 2002). With increasing task load and sustained requirements for updating and monitoring, the DLPFC is more strongly recruited and operates in coordination with parietal regions to support accurate performance under high demand. Accordingly, θ-tACS targeting the DLPFC is theoretically well positioned to modulate the relevant rhythms and control network. EEG findings further demonstrate that DLPFC θ oscillations increase under higher working memory load, accompanied by enhanced parietal synchrony, highlighting the functional importance of prefrontal θ activity for maintaining executive control (Fernández et al., 2021). In parallel, n-back fMRI-DCM studies show that forward effective connectivity from the left DLPFC to parietal regions strengthens with increasing load and is positively associated with task accuracy, supporting the left DLPFC as an upstream node within the executive control network during high-load processing (Heinzel et al., 2017; Jung et al., 2018). In line with these observations, we found a significant post-stimulation increase in task-evoked β values in the left DLPFC following 6 Hz tACS, and this change was positively correlated with improvements in 3-back accuracy; no comparable effects were observed in the 1-back or 2-back conditions, suggesting load-dependent selectivity for executive processing.

### 4.3 Network-level interpretation and bilateral stimulation rationale

Furthermore, many prior stimulation studies have adopted unilateral or lateralised DLPFC montages, targeting either the left or right hemisphere depending on task demands. However, converging neuroimaging evidence indicates that high-load working memory robustly engages bilateral DLPFC as part of the frontoparietal control network; for example, longitudinal fMRI work using high-load contrasts 2−*back*/3−*backvs*.0−*back* has shown reliable bilateral DLPFC recruitment (Miró-Padilla et al., 2020). Motivated by this network organization, bilateral (or multi-node) stimulation may better support demanding working-memory operations by promoting interhemispheric coordination and large-scale network integration. In line with this, Nissim et al. combined bilateral frontal tDCS (F3/F4) with fMRI and observed stimulation-related modulation of working-memory network connectivity in healthy older adults (Nissim et al., 2019). Similarly, bifrontal protocols have been reported to yield sustained behavioral benefits, including multi-session prefrontal stimulation approaches in older adults (Diedrich et al., 2025) and bifrontal tACS effects on verbal working memory in young adults (Raviv et al., 2025). Together, these findings provide external support for our bilateral 6 Hz tACS approach, which aims to co-engage both DLPFCs and thereby facilitate network-level coordination rather than modulating a single cortical locus.

### 4.4 fNIRS haemodynamic signatures and lateralization

Although we did not directly quantify θ power or phase synchrony, enhanced recruitment of rhythmic networks can manifest downstream via neurovascular coupling as a stronger task-locked haemodynamic response, making fNIRS a complementary window into these effects. Within the fNIRS-GLM framework, β values reflect the amplitude of task-evoked changes in oxygenated hemoglobin (HbO) relative to baseline and can be interpreted as a macroscopic indicator of neurovascular coupling (NVC) (Zhang and Xu, 2025). Accordingly, larger β values indicate a stronger task-locked HbO response in the relevant cortical region. In the present study, the 6 Hz group exhibited a significant increase in left DLPFC β values during the high-load 3-back condition, and this increase correlated positively with accuracy gains, suggesting that the behavioral benefit of 6 Hz tACS may reflect augmented recruitment of left prefrontal executive control resources under high load, leaving a detectable haemodynamic signature. Notably, the haemodynamic enhancement was predominantly left-lateralised. We propose that this discrepancy may reflect the combined influence of task material characteristics and frequency matching. Digit-based N-back tasks are often performed using subvocal rehearsal, aligning more closely with verbal/symbolic working memory and thereby placing greater demands on left-hemispheric prefrontal representational and operational resources; under high load, this left-sided control demand may further increase. This interpretation should nevertheless be made cautiously, because the present design did not include a spatial working-memory condition. Future studies could incorporate a spatial N-back paradigm, or directly compare verbal/symbolic and spatial task materials, to determine whether the observed left-lateralised haemodynamic modulation is task-specific or reflects a more general pattern of hemispheric lateralisation in working-memory control. Meanwhile, 6 Hz θ-tACS aligns with the prefrontal θ band commonly engaged during working memory processing and may therefore more effectively entrain or align rhythms within the high-load window, enhancing left DLPFC top-down regulation and network coordination, which is expressed haemodynamically as increased β values (Yeh et al., 2023; Pahor and Jaušovec, 2017). Thus, the observed left DLPFC predominance not only supports a “left-biased control” account for verbal/symbolic working memory, but also suggests that future optimisation of θ-tACS protocols should consider task material and load state: under verbal paradigms, the left DLPFC may provide a more sensitive stimulation target and imaging marker, offering testable explanations for heterogeneity in lateralised vs. bilateral activation patterns across studies.

### 4.5 Limitations and future directions

Several limitations should be noted. First, the sample size was modest and restricted to healthy young adults, which may limit generalisability to other age groups or clinical populations. Second, only a single stimulation session was tested; future multi-session studies with follow-up assessments are needed to determine durability and dose–response relationships. Third, although sham stimulation was designed to mimic the cutaneous sensations associated with active stimulation and thereby support single blinding, we did not include a formal post-stimulation assessment of whether participants could distinguish active from sham stimulation. Fourth, we did not directly measure electrophysiological indices of theta entrainment or interhemispheric synchrony; multimodal designs combining EEG with fNIRS would help link oscillatory mechanisms to haemodynamic changes. Finally, fNIRS is primarily sensitive to superficial cortical layers and remains vulnerable to systemic physiological confounds despite preprocessing, so deeper and whole-brain network effects could not be assessed.

## 5 Conclusion

Bilateral 6 Hz tACS targeting the DLPFC selectively improved high-load working memory, as reflected by greater gains in 3-back accuracy and Digit Span performance compared with sham. These behavioral improvements were accompanied by increased task-evoked haemodynamic responses in the left DLPFC and a positive brain–behavior association, suggesting that theta-frequency stimulation may enhance prefrontal executive control under high cognitive demand. Future studies should test multi-session protocols, include electrophysiological measures to verify entrainment, and extend validation to clinical populations to establish translational relevance.

## Data Availability

The original contributions presented in the study are included in the article/supplementary material, further inquiries can be directed to the corresponding author/s.
